# Application of GC/MS-Based Metabonomic Profiling in Studying the Therapeutic Effects of *Aconitum carmichaeli* with *Ampelopsis japonica* Extract on Collagen-Induced Arthritis in Rats

**DOI:** 10.3390/molecules24101934

**Published:** 2019-05-20

**Authors:** Hua Jin, Ningning Ma, Xin Li, Mingqin Kang, Maojuan Guo, Lili Song

**Affiliations:** 1 College of Traditional Chinese Medicine, Tianjin University of Traditional Chinese Medicine, Jian Kang Chan Ye Yuan, Jinghai Dist., Tianjin 301617, China; zyxyjin@163.com; 2 School of Traditional Chinese Materia Medica, Tianjin University of Traditional Chinese Medicine, Jian Kang Chan Ye Yuan, Jinghai Dist., Tianjin 301617, China; mningning646594@163.com (N.M.); lixin9563@sina.com (X.L.); 3 Changchun Customs (Former Jilin Inspection and Quarantine Bureau), Changchun 130012, China; fjjonup@163.com; 4 Department of Pathology, School of integrative Medicine, Tianjin University of Traditional Chinese Medicine, Jian Kang Chan Ye Yuan, Jinghai Dist., Tianjin 301617, China

**Keywords:** GC-MS, rheumatoid arthritis, *Aconitum carmichaeli* with *Ampelopsis japonica* (AA), urine metabonomics

## Abstract

*Aconitum carmichaeli* with *Ampelopsis japonica* (AA) is a classical traditional Chinese medicine (TCM) formula. There are a lot of examples showing that AA can be used to treat rheumatoid arthritis, but its mechanism of action is still not completely clear. In this research, collagen-induced arthritis (CIA) was chosen as a rheumatoid arthritis (RA) model. Rats of treated groups were continuously administered *Aconitum carmichaeli* (AC), *Ampelopsis japonica* (AJ) and *Aconitum carmichaeli* + *Ampelopsis japonica* (AA) orally once a day from the day after the onset of arthritis (day 7) until day 42. The results showed that AA not only significantly reduced paw swelling, but also improved the levels of TNF-α and IL-6 in serum. GC-MS-based urine metabonomics was established to analysis metabolic profiles and 21 biomarkers of RA rats were identified by the Partial Least Squares Discriminant Analysis (PLS-DA) and Support Vector Machine (SVM) methods. The prediction rate of the SVM method for the 21 biomarkers was 100%. Twenty of 21 biomarkers, including D-galactose, inositol and glycerol, gradually returned to normal levels after administration of AA. Metabolomic Pathway Analysis (MetPA) generated three related metabolic pathways—galactose metabolism, glycerolipid metabolism and inositol phosphate metabolism—which explain the mechanism of AA treatment of rheumatoid arthritis. This research provides a better understanding of the therapeutic effects and possible therapeutic mechanism of action of a complex TCM (AA) on rheumatoid arthritis.

## 1. Introduction

Rheumatoid arthritis (RA) is an autoimmune disease that affects approximately 1% of people worldwide [1]. Environmental factors, such as smoking, and genetic factors are the causes of rheumatoid arthritis. The genetic factors are reported to be the main cause of rheumatoid arthritis, accounting for 50% [2]. RA is also a common inflammatory disease, often affecting other organs or systems, such as synovial joints [3], and causing interstitial lung disease [4], and nervous system injuries [5]. The joints of RA patients are swollen and gradually become stiff, affecting the action and causing inconvenience [6]. With the gradual development of modern medicine, treatments for RA have also been studied [7]. However, a more effective treatment of RA is still an urgent necessity for modern medicine [8,9].

*Aconitum carmichaeli* with *Ampelopsis japonica* (AA) is a classical traditional Chinese medicine (TCM) formula. It was composed of *Aconitum carmichaeli* and *Ampelopsis japonica*. *Aconitum carmichaeli*, a traditional Chinese medicine, has been used for the treatment of rheumatism and joint pain since ancient times in China [10]. A study has shown that 75% ethanol extracts of *Aconitum carmichaeli* has anti-arthritic effect on arthritis induced by Freund’s complete adjuvant in rats [11]. *Ampelopsis japonica* has an anti-inflammatory effect and can regulate inflammatory factors such as TNF-α and IL-10 [12]. In ancient clinical applications, this formula was first described in “Da Ba Feng San” by Sun Si-miao (in the Chinese Tang Dynasty) in his “QianJinFang” [13]. Ming et al. used a traditional Chinese medicine fumigation method containing *Aconitum carmichaeli* and *Ampelopsis japonica* to treat knee osteoarthritis with remarkable curative effect, and the cure rate was 96.55% [14]. Cheng et al. used these two and other traditional Chinese medicines combined with sodium hyaluronate to treat arthritis. Treatment results showed that the patients’ joint swelling was eliminated and the pain relieved [15]. Since then, AA has been widely used for the treatment of rheumatoid arthritis in China. Our previous studies have indicated that AA has therapeutic potential in rheumatoid arthritis treatment; however the specific mechanism of action remains unclear.

In recent years, due to its concentration on internal metabolites in organisms, metabolomics has been widely used in the study of disease mechanisms and metabolic changes by discovering potential biomarkers and associated metabolic pathways [16,17,18]. Metabonomics can analyze the physiological and pathological changes of the whole biological system, which is holistic and comprehensive. At present, the combination of GC/MS technology and metabonomics analysis is widely used for screening and diagnosis of disease markers [19,20,21]. Support Vector Machine (SVM), a classification metho based on supervised learning and binary classification, plays an important role in metabonomics analysis [22,23]. SVM is mainly used for further screening and identification of biomarkers [24,25]. Metabolomics data is various and complicated. Multivariate statistical analysis is an effective statistical analysis distribution, which facilitates the visualization of multidimensional and complex data [26]. In general, multivariate statistical variable analysis is mostly based on SIMCA-P software and is widely used in multivariate statistical analysis of food [27,28], traditional Chinese medicines and prescriptions [29,30,31], and disease metabonomics studies [32].

In this experiment, female Sprague Dawley (SD) rats were randomly divided into five groups: normal control (NC), RA model (RA), *Aconitum carmichaeli*-treated RA (AC), *Ampelopsis japonica*-treated RA (AJ) and *Aconitum carmichaeli* + *Ampelopsis japonica*-treated RA (AA). We utilized collagen-induced arthritis in rats as the rheumatoid arthritis model. The hind paw swelling of rats, serum TNF-α and IL-6 levels were measured to find out the group that showed better therapeutic effects in the RA rats. GC/MS-based metabonomics was developed to explore the changes of metabolic profiles in the urine samples from different groups. We aim at finding out and identifying the specific biomarkers of RA via multivariate statistical analysis, and exploring the possible therapeutic mechanism of AA for RA.

## 2. Results

### 2.1. Results of Hind Paw Volume and Biochemical Parameters

**Figure 1:**
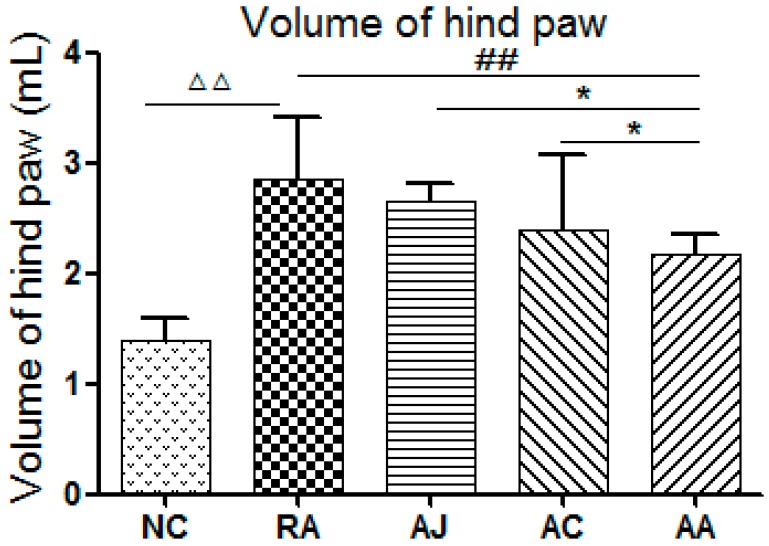
Hind paw volumes of the rats. (NC, control group; RA, model group; AC, *Aconitum carmichaeli*-treated RA group; AJ, *Ampelopsis japonica*-treated RA group; AA, *Aconitum carmichaeli* + *Ampelopsis japonica*-treated RA group, ^△△^ *p* < 0.01 RA vs. NC; ^##^ *p* < 0.01 AA vs. RA; * *p* < 0.05 AC/AJ vs. AA).

Hind paw swelling of RA rats was observed obviously. Compared with the NC group, the hind paw volume in RA group increased significantly (*p* < 0.01) (Figure 1). After TCM treatment, the AC and AJ groups displayed slight inhibitory effects on paw swelling. However, the AA had a marked suppression effect on RA rats (*p* < 0.05), compared with the NC group (Figure 1).

**Figure 2:**
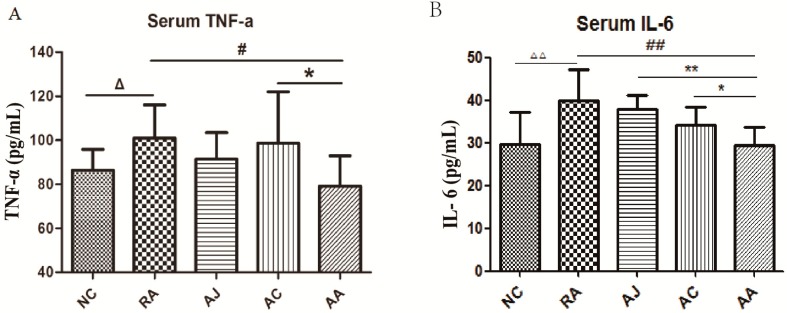
Effects of *Aconitum carmichaeli*, *Ampelopsis japonica* and their combination on serum TNF-α (**A**) and IL-6 (**B**) levels of the RA rat. Data is expressed as mean ± SEM (*n* = 12). (NC, control group; RA, model group; AC, *Aconitum carmichaeli*-treated RA group; AJ, *Ampelopsis japonica*-treated RA group; AA, *Aconitum carmichaeli* + *Ampelopsis japonica*-treated RA group). ^△^ *p* < 0.05 RA vs. NC; ^△△^ *p* < 0.01 RA vs. NC; ^#^ *p* < 0.05 AA vs. RA: ^##^ *p* < 0.01 AA vs. RA; * *p* < 0.05 AA vs. AC, ** *p* < 0.01 AA vs. AJ.

On day 42 blood samples were collected. The serum IL-6 and TNF-α levels were determined. (Figure 2A,B, respectively). The serum TNF-α and IL-6 levels were higher in the model group than in the normal group (*p* < 0.05). The AA group showed lower TNF-α and IL-6, and the AC and AJ groups had no obvious effect on TNF-α and IL-6. This showed that the anti-inflammatory effect of the combination was better than that of the single drug. Therefore, we chose the combination group for the following metabolomics studies.

### 2.2. Analysis Results of GS-MS

**Figure 3:**
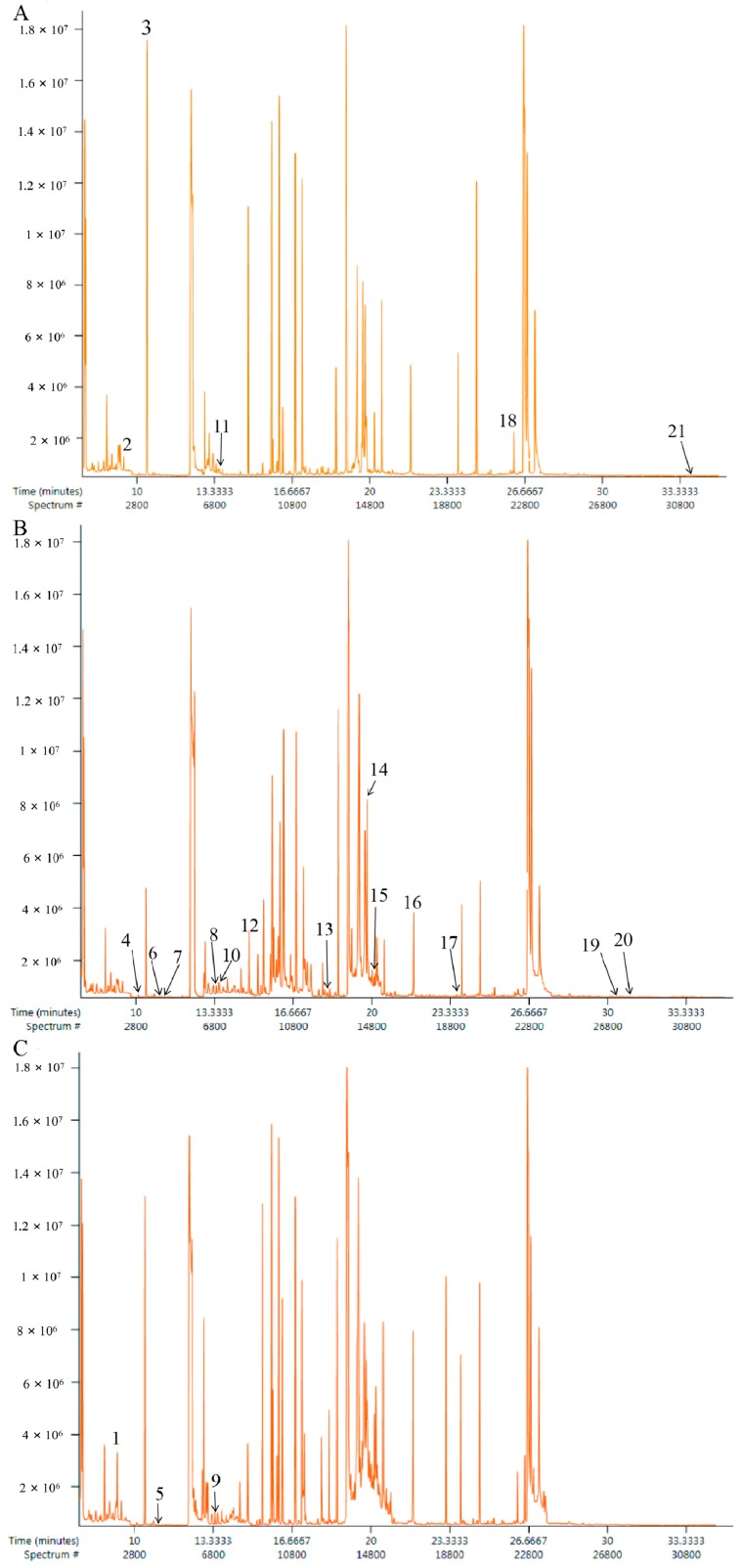
GC/MS chromatograph of urine samples obtained from the NC group (**A**), RA group (**B**) and AA group (**C**), respectively.

Typical total ion chromatograms (TIC) of urine samples from the NC, RA, and AA groups are presented in Figure 3.

### 2.3. Metabolic Profiling for RA Rats

#### 2.3.1. Biomarker Identification

**Figure 4:**
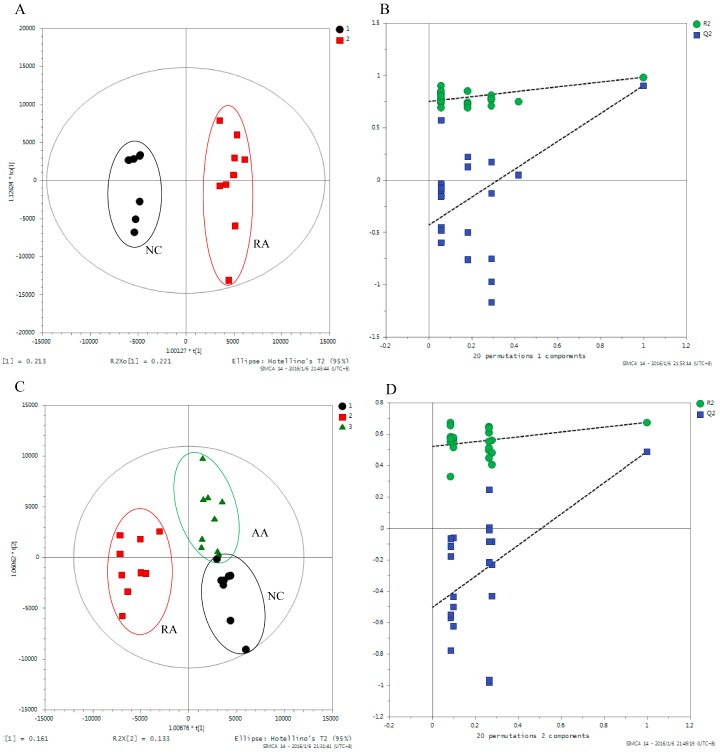
PLS-DA score plot and validation of PLS-DA model of urine samples from the rats. (**A**) PLS-DA score plot of NC and RA group; (**B**) validation of PLS-DA model of NC and RA group; (**C**) PLS-DA score plot of NC, RA and AA group; (**D**) validation of PLS-DA model of NC, RA and AA group. (NC represents control group; RA represents model group; AA represents the *Aconitum carmichaeli* + *Ampelopsis japonica*-treated RA group).

The metabolic profile analysis was performed using SIMCA-P ^+^ (Version 14.0, Umetrics, Umea, Sweden). Figure 4A shows the score plot of PLS-DA (R^2^Y = 0.982, Q^2^ = 0.902) based on the NC group and the RA group. The RA group was clearly separated from NC group, which illustrated RA seriously disturbed the normal metabolism in rats and RA model was developed successfully. We selected metabolites with VIP > 1 (PLS-DA) and *p* < 0.05 (*t* test) as potential biomarkers. Based on the above range of values, a total of 21 differential metabolites which caused metabolic disturbance in the RA group were screened out in rat urine (Table 1). Compared with the NC group, RA rats display increased levels of 15 metabolites including 3-(allyloxy)propanoic acid, 4-ketoglucose, glycerol, myoinositol and so on; and the levels of six metabolites decreased, including D-galactose, *p*-cresol, propanal and so on (Table 1). The result of permutation tests indicated that the establishment for PLS-DA model is reliable without overfitting (Figure 4B).

**Figure 5:**
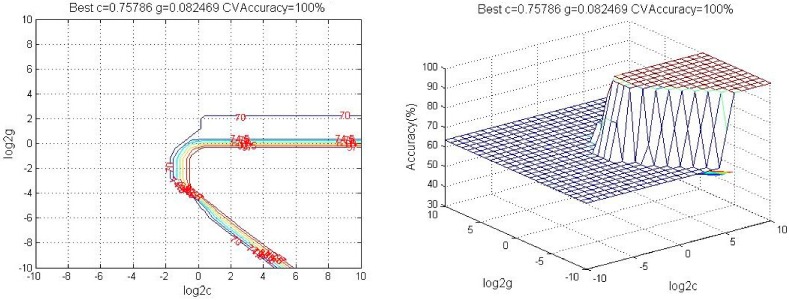
A 3D view of the SVM model of 21 biomarkers (The parameters are described below: Best c = 0.75786, g = 0.082469, CV accuracy = 100%, 112 × 86 mm (123 × 123 DPI).

Then we classified the potential biomarkers using SVM. The data from all 36 samples were randomly divided into the training set (24 samples) and the test set (12 samples), respectively. The prediction accuracy rate of the model established by the potential biomarkers for the prediction of rheumatoid arthritis was 100% (Figure 5, Best c = 0.75786, Best g = 0.082469, CV accuracy = 100%).

#### 2.3.2. Target Analyses of Highlighted Metabolites of AA Treatment

**Figure 6:**
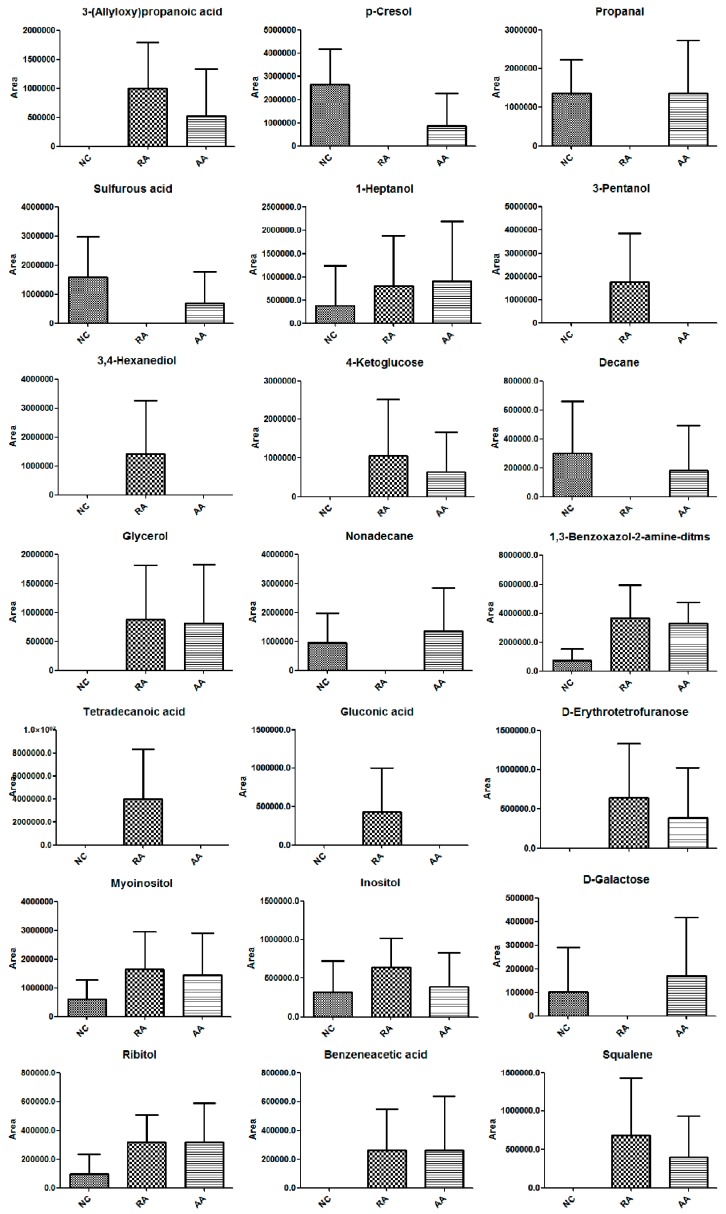
Areas of chromatographic peak of 21 biomarkers in NC, RA and AA groups.

A separate PLS-DA model (R^2^Y = 0.734, Q^2^ = 0.568) can discriminate the RA and AA group (Figure 4C). The samples of group AA (green triangles) and group RA (red squares) had a distinct separation trend, and gradually approached the NC group (black dots). The results showed that the metabolic spectrum of RA rats began to recover to normal levels after treatment with AA. The validation plot supported the validity of this PLS-DA model (Figure 4D). Twenty of the 21 metabolites showed opposite trends in RA group and AA group (Table 1), which means that the metabolism of these metabolites has a tendency to return to normal level after AA intervention. Areas of chromatographic peaks of 21 biomarkers in NC, RA and AA group were showed in Figure 6. Therefore, these 20 metabolites are ultimately screened out as specific biomarkers about AA treatment of rheumatoid arthritis.

### 2.4. Mechanism Analysis of AA Treatment RA Rats

**Figure 7:**
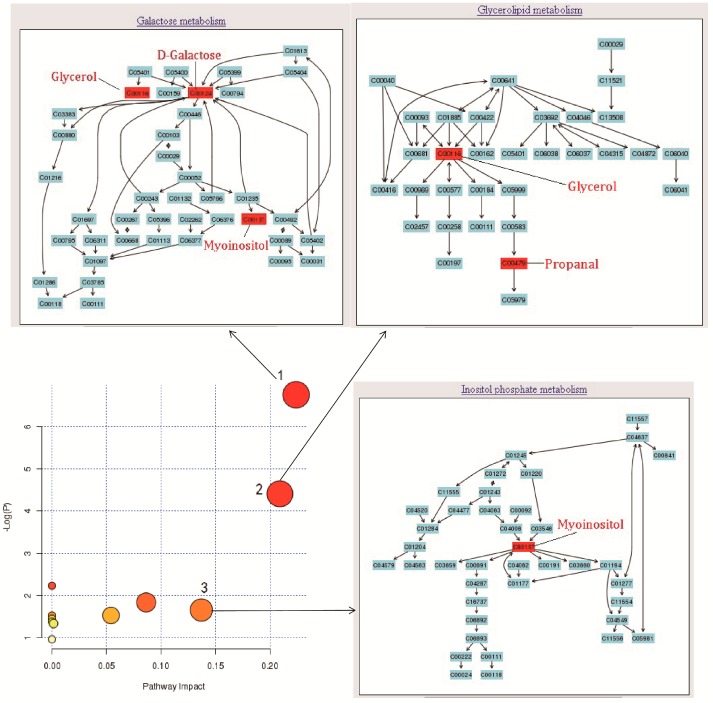
Summary of pathway analysis: (1) galactose metabolism; (2) glycerolipid metabolism; (3) inositol phosphate metabolism.

Metabolomic Pathway Analysis (MetPA) is a database visualizing metabolic pathway analysis (www.metaboanalyst.ca). In this experiment, 11 networks were obtained with MetPA (Table 2). The threshold of impact was set to 0.10. The pathway is considered to be a closely related if its impact value is higher than 0.1. Finally, three underlying pathways, galactose metabolism, glycerolipid metabolism and inositol phosphate metabolism, were filtered out, which were significant for treatment of AA for rheumatoid arthritis (Figure 7).

## 3. Discussion

**Figure 8:**
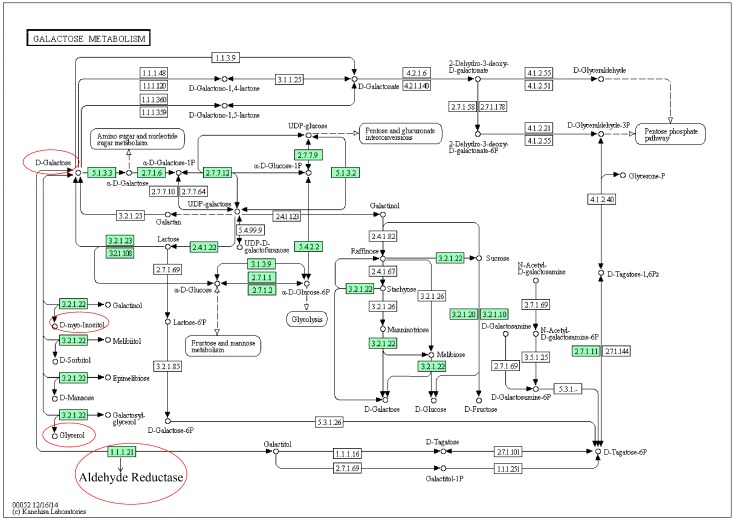
Construction of the galactose metabolism. The map was generated using the reference map by KEGG (http://www.genome.jp/kegg/).

D-Galactose, glycerol and myoinositol were selected as the differential markers, and the correlation analysis showed that they belonged to galactose metabolism (Figure 7). D-Galactose was significantly decreased, whereas glycerol and myoinositol were significantly increased in the RA inflammation model group, and AA group was able to return these metabolites to normal levels. In the RA group, the D-galactose level was significantly lower than that in the control group, while in the AA group it was significantly increased. Galactose metabolism is usually caused by the action of A-galactosidase on glucose and galactose [33], and under RA stimulation, there is more conversion of galactose to glucose, therefore, the content of galactose in urine was significantly reduced. The AA group can improve the galactose content level, making the galactose metabolism normal. Galactose metabolism and glycolysis metabolic pathways are closely related. Galactose generates 1-phosphate galactose after phosphorylation, then uridine diphosphate galactose (UDP-galactose) catalyzed by phosphate transferase enzyme galactose uridine and UDP-galactose C4-isomer generated UDP-glucose, which in the liver with galactose is routed to glucose metabolic pathways [34]. The overall metabolic network structure of galactose metabolism is shown in Figure 8.

Glycerol and propanal were selected as differential markers, and the correlation analysis showed that they belonged to the glycerolipid metabolism pathway (Figure 8). Glycerol was significantly increased, while propanal was significantly decreased in the RA inflammation model group, the AA group was able to return these metabolites to normal values. Lipid metabolism is very important in the occurrence and development of the disease. Changes in lipid metabolites can lead to a series of pathophysiological phenomena, including obesity [35,36], type 2 diabetes (T2D) [37], inflammation [38], non-alcoholic fatty liver disease, cancer, etc. [39]. The results of this experiment showed that the different metabolites of glycerol affected glycerolipid metabolism, and the glycerol level was significantly increased in the RA model group, and in the AA group it was significantly higher than that in the control group. With RA stimulation, impaired lipid metabolism occurs and liver X receptor gene expression will be reduced by inflammation [40]. Studies have shown that liver X receptor can be oxidized by lipid molecules, such as fatty acid oxidation products and metabolites of arachidonic acid [41]. These potential activators derive from unsaturated fatty acids, cholesterol and its metabolites in some basic metabolic processes, especially triglycerides, as the balance of triglycerides in the body and the conversion between glycerol esters and fatty acids have a great relationship. These results suggest that the reason glycerol is increased in the RA inflammatory group is the destroyed mutual transformation between glycerol and fatty acids, leading to lipid metabolism disorders, thus reducing the liver X receptor expression, whereas AA can reduce the content of glycerol, rebalancing the fatty acid glycerides and mutual transformation balance, and causing liver X receptor upregulation in order which results in an anti-inflammatory effect.

Inositol was selected as the differential marker, and the correlation analysis showed that it belonged to the inositol phosphate metabolism pathway (Figure 8). Myoinositol was significantly increased in the RA inflammation model group and the AA group was able to return to nirmal the levels of these metabolites. Inositol is a cyclic polyalcohol that plays an important role as a second messenger in a cell, in the form of inositol phosphates [42]. Nearly 20 different kinds of inositol phosphates have been found so far, which have been found to have an important role as information material. The most important is inositol triphosphate (IP3), which is triphosphoinositide [43]. Its main function is to induce Ca^2+^ release from the cell, and increase the concentration of Ca^2+^ in the cell. The elevation of intracellular calcium concentration is a necessary condition for the activation of some inflammatory cells, including neutrophils, as well as the activation of the related enzymes and release of inflammatory mediators [44]. The existing data shows that regulating the Ca^2+^ concentration in the neutrophil cytoplasm is an effective way to regulate the inflammatory response [45].

## 4. Materials and Methods

### 4.1. Materials

*Aconitum carmichaeli* and *Ampelopsis japonica* (Anguo Putianhe Traditional Chinese Medicine Co., Ltd., Anguo, China) were identified as the dried taproot of *Aconitum carmichaeli* Debx, and dried rhizome of *Ampelopsis japonica* (Thunb.) Makino, respectively, by Associate Professor Tianxiang Li. *Aconitum carmichaeli* and *Ampelopsis japonica* (1 kg, respectively) were refluxed three times with water (10 times volume) for 1 h each time. The filtrates were combined and concentrated. The dosage for rats was 0.6 g crude drug per 100 g body weight daily. All rats were treated by once daily intragastric administration with normal saline or herbs, respectively, for 5 weeks from the day after the onset of arthritis (day 7).

Incomplete Freund’s adjuvant [(IFA), Chondrex, 7002, Redmond, WA, USA], type II collagen [(CII), Chondrex, 2002,], distilled water was prepared with a Milli-Q Reagent Water System (Millipore, Bedford, MA, USA). LC-grade acetonitrile, bis(trimethylsilyl) trifluoroacetamide (BSTFA) and trimethylchlorosilane (TMCS) were obtained from Regis Technologies (Inc., Morton Grove, IL, USA). l-2-Chlorophenylalanine (CAS#: 103616-89-3, 98%) was purchased from Shanghai Hengbai Biotechnology Co., Ltd. (Shanghai, China), Methanol was of analytical grade.

### 4.2. Animals and Sample Preparation

A total of 60 female Sprague Dawley (SD) rats with body weights of 170 ± 10 g were purchased from the Chinese PLA Institute of Hygiene and Environmental Medicine of the Military Medical Science Academy of the PLA. The rats were kept in a controlled environment at specific-pathogen free (SPF) levels and were allowed to access food and water ad libitum. All animal experiments were approved by Animal Ethics Committee of Tianjin University of Traditional Chinese Medicine (TCM-2012-010-E01).

CIA model was induced by a modification of the method described in [46]: in brief, type II collagen was dissolved in acetic acid (0.05 M) and emulsified with an equal volume of IFA, and the homogenate was fully emulsified in the ice bath, until the solution is not spread with water. A 0.2 mL emulsion sample was injected intracutaneously into the tail of each rat, 2 cm from the base. Seven days after the first injection, a 0.2 mL emulsion sample was injected as a booster. After this second immunization, animals with were randomly divided to model and treatment groups respectively. The normal group was injected the volume of saline in accordance with the above method were used as a control group.

### 4.3. Physical Parameters

**Figure 9:**
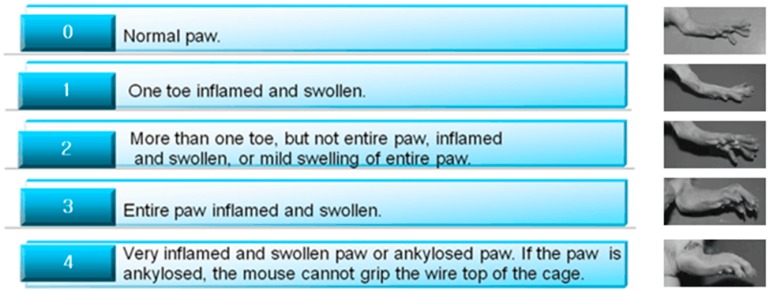
Paw score of the CIA in the clinical observations.

Body weight and hind paw volume were determined weekly. The hind paw volumes were measured with a plethysmometer (YLS-7B, Beijing Kanghuishengda Technology Co., Ltd., Beijing, China), and the hind paw volume was the average of both hind paws. Arthritis score of the limbs were evaluated by two independent, blinded observers following the criteria shown in Figure 9. When the highest score of both hind paws was 4 for each animal, we chose it as a model.

### 4.4. Measurements of Serum TNF-α, IL-6 Levels

All rats were sacrificed to collect blood for biochemical analysis at the end of experiment. The corresponding ELISA kits (Baomanbio Co., Ltd., Shanghai, China) were used to measure levels of TNF-α and IL-6 in serum.

### 4.5. Urine Sample Preparation

**Figure 10:**
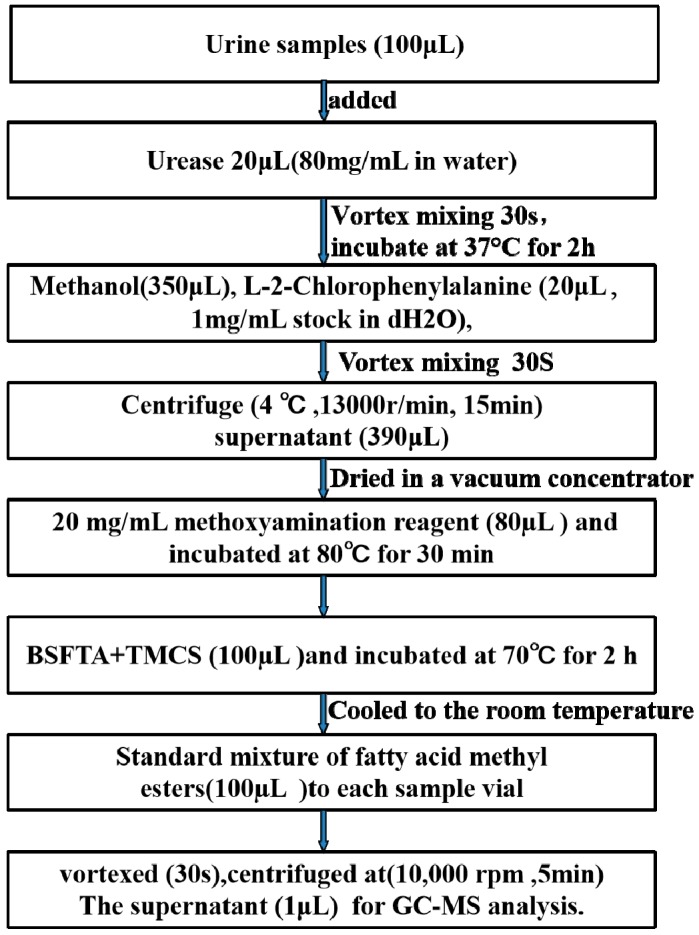
The urine sample flow- processing.

Urine samples were collected from rats for 12 h before rats were sacrificed. The urine samples were centrifuged at 4 °C for 10 min. All supernatant was stored at −80 °C until measurement. The urine samples were thawed at room temperature before using. The sample processing was in Figure 10.

### 4.6. GC-MS Analysis Conditions

Samples were injected into the GC-MS (Agilent 7890, Agilent, Palo Alto, CA, USA) system equipped a DB-5MS capillary column (Agilent 0.25 mm × 30 m × 0.25 μm, J&W Scientific, Folsom, CA, USA) and FID detector. The volume of injection was 1 μL, no streaming. Helium (99.9996%) was used as the carrier gas, the front inlet purge flow was 3 mL min^−1^, and the gas flow rate through the column was 1 mL min^−1^. The initial temperature was kept at 50 °C for 1 min, then raised to 300 °C at a rate of 10 °C min^−1^, then kept for 9 min at 300 °C. The injection, transfer line, and ion source temperatures were 280, 270, and 220 °C, respectively. The energy was −70 eV in electron impact mode. The mass spectrometry data were acquired in full-scan mode with the m/z range of 50–500 at a rate of 20 spectra per second after a solvent delay of 460 s.

### 4.7. Date Analysis

SPSS 17.0 statistical software (SPSS Inc., Chicago, IL, USA) was used for statistical analysis. Data were expressed as mean ± standard deviation (SD). The *t* test was used to calculate the significance *P* (*P* < 0.05 is of statistical significance).

The common peaks are selected according to the retention time of each peak in the total ion chromatogram of GC-MS, which is based on the principle that only the peaks detected in 80% of the samples can be selected. The NIST customized reference mass spectral libraries and the mass spectral library (Wiley Registry, 2008 edition) [47] was used to determine all detected peaks by similarity matching (similarity > 80%). After the above processing, a three-dimensional matrix was obtained, including the specified peak index (retention time—*m*/*z*), sample names and peak areas.

Partial least squares discriminant analysis (PLS-DA) is used to select the differential metabolites from two groups (VIP >1) combined with *p*-value < 0.05 (*t* test). Cross-validation can detect if the model established by the pattern recognition method is over-fitting [48,49,50]. The SVM method could diagnose the sample classification ability of pattern recognition methods and provide a quantitative assessment of the classification ability. Finally, we determined the prediction accuracy rate by mechanical training with testing and training sets.

## 5. Conclusions

In summary, the GC-MS technique was used to explore the metabonomic effects of AA extract on collagen-induced arthritis in rats. The physical and biochemical parameter measurement results suggested that AA treatment has a better therapeutic effect on RA than AC and AJ treatments. Twenty one metabolites were identified as potential biomarkers of RA rats by metabolic profile analysis of urine. The metabolomic results also illustrated that AA possess a therapeutic effect on RA through partially regulating the perturbed galactose metabolism, glycerolipid metabolism and inositol phosphate metabolism. Finally, twenty specific biomarkers and three related metabolic pathways of AA treatment of RA were identified in our study, which should be helpful for interpreting the metabolic effects of rheumatoid arthritis and the mechanisms of action of AA against rheumatoid arthritis. This research demonstrates novel perspectives for confirmation and verification of biomarkers via metabolomics in rheumatoid arthritis and provides a better understanding of evaluating the therapeutic effects and mechanism of complex TCM (AA) treated on the rheumatoid arthritis.

## 6. Limitations

Nevertheless, there are still some disadvantages in this study. Although biomarkers and metabolic pathways have been identified, their association with AA treatment of RA is not well understood. Experiments should be continued and further research needs to be done.

## Figures and Tables

**Table 1:** Differential metabolites were detected by GC/MS and their metabolic pathways.

No.	R.T. (min)	Metabolite	Match Percent/%	Related Pathway	Trend	
					RA Group ^a^	AA Group ^b^
1	8.647	3-(Allyloxy)propanoic acid	68		↑	↓
2	9.385	*p*-Cresol	87		↓	↑
3	10.301	Propanal	75	Glycerolipid metabolism	↓	↑
4	10.705	Sulfurous acid	76		↓	↑
5	11.194	1-Heptanol	88		↑	↑
6	11.402	3-Pentanol	82		↑	↓
7	11.721	3,4-Hexanediol	62		↑	↓
8	13.401	4-Ketoglucose	85		↑	↓
9	13.507	Decane	94		↓	↑
10	13.554	Glycerol	80	Glycerolipid metabolism	↑	↓
11	13.601	Nonadecane	94		↓	↑
12	14.421	1,3-Benzoxazol-2-amine-ditms	80		↑	↓
13	18.162	Tetradecanoic acid	94		↑	↓
14	19.803	Gluconic acid	78		↑	↓
15	19.916	d-Erythrotetrofuranose	82		↑	↓
16	21.916	Myoinositol	82	Inositol phosphate metabolism	↑	↓
17	23.369	Inositol	79	Inositol phosphate metabolism	↑	↓
18	26.353	d-Galactose	77	Galactose metabolism	↓	↑
19	30.818	Ribitol	78		↑	↓
20	31.326	Benzeneacetic acid	66		↑	↓
21	33.779	Squalene	86		↑	↓

↑: the compound is up-regulated, ↓: the compound is down-regulated, ^a^ Change trend: RA group compared with NC group, ^b^ Change trend: AA group compared with RA group.

**Table 2:** Results from Pathway Analysis with MetPA.

No.	Pathway Name	Total	Expected	Hits	Raw *p*	Holm *p*	FDR *p*	Impact
1	Galactose metabolism	41	0.22	3	1.17 × 10^−6^	9.33 × 10^−2^	9.33 × 10^−2^	0.22
2	Glycerolipid metabolism	32	0.17	2	1.22 × 10^−2^	9.63 × 10^−1^	4.88 × 10^−1^	0.21
3	Riboavin metabolism	21	0.11	1	1.08 × 10^−1^	1.00	1.00	0.00
4	Pentose phosphate pathway	32	0.17	1	1.60 × 10^−1^	1.00	1.00	0.09
5	Inositol phosphate metabolism	39	0.21	1	1.92 × 10^−1^	1.00	1.00	0.14
6	Ascorbate and aldarate metabolism	45	0.24	1	2.18 × 10^−1^	1.00	1.00	0.00
7	Phenylalanine metabolism	45	0.24	1	2.18 × 10^−1^	1.00	1.00	0.05
8	Fatty acid biosynthesis	49	0.26	1	2.35 × 10^−1^	1.00	1.00	0.00
9	Pentose and glucuronate interconversions	53	0.29	1	2.52 × 10^−1^	1.00	1.00	0.00
10	Cysteine and methionine metabolism	56	0.30	1	2.64 × 10^−1^	1.00	1.00	0.01
11	Amino sugar and nucleotide sugar metabolism	88	0.48	1	3.85 × 10^−1^	1.00	1.00	0.02

Total is the total number of compounds in the pathway; the Hits is the actually matched number from the user uploaded data; the Raw *p* is the original *p* value calculated from the enrichment analysis; the Holm *p* is the *p* value adjusted by Holm-Bonferroni method; the FDR *p* is the *p* value adjusted using False Discovery Rate; the Impact is the pathway impact value calculated from pathway topology analysis.
